# Viral Risks at the Human–Bat Interface: Household Bat Guano Farming in Rural Cambodia

**DOI:** 10.3390/pathogens15050485

**Published:** 2026-04-30

**Authors:** Theara Teng, Sarin Neang, Bruno M. Ghersi, Cora Cunningham, Daniel Nguyen, Felicia B. Nutter, Veasna Duong, Thavry Hoem, Sothyra Tum, Theary Ren, Dina Koeut, Sam Eang Huon, Sothealy Oeun, Jonathon D. Gass, Janetrix Hellen Amuguni, Daniele Lantagne, Tristan L. Burgess

**Affiliations:** 1 TetraTech ARD, Phnom Penh Center, Sangkat Tonle Basac, Khan Chamkar Mon, Phnom Penh 12301, Cambodia; neangsarin@gmail.com; 2 Cummings School of Veterinary Medicine, Tufts University, North Grafton, MA 01536, USA; bruno.ghersi_chavez@tufts.edu (B.M.G.); felicia.nutter@tufts.edu (F.B.N.); tburgess@centerforwildlifestudies.org (T.L.B.); 3 Department of Global Health and Population, Harvard T.H. Chan School of Public Health, Boston, MA 02115, USA; coracunningham@hsph.harvard.edu; 4 School of Medicine, Tufts University, Boston, MA 02111, USA; nguyen.dan@tufts.edu (D.N.); jonathon.gass@tufts.edu (J.D.G.); janetrix.amuguni@tufts.edu (J.H.A.); 5 Institut Pasteur du Cambodge, Phnom Penh P.O. Box 983, Cambodia; dveasna@gmail.com (V.D.); hoemthavry1@gmail.com (T.H.); 6 National Animal Health and Production Research Institute, Khan Mean Chey, Phnom Penh 120603, Cambodia; sothyratum@gmail.com (S.T.); rentheary2020@gmail.com (T.R.); koeutdina9@gmail.com (D.K.); 7 Forestry Office, Provincial Department of Agriculture, Forestry and Fisheries, Kampong Cham, Cambodia; sameanghuon@gmail.com; 8 Kang Meas District Authority, Kampong Cham, Cambodia; sothealy7567@gmail.com; 9 Feinstein International Center, Friedman School of Nutrition Science and Policy, Tufts University, Boston, MA 02111, USA; daniele.lantagne@tufts.edu; 10 Center for Wildlife Studies, Camden, ME 04843, USA

**Keywords:** bat roost, coronaviruses, guano, surface contamination, viral spillover

## Abstract

In Cambodia, farmers construct artificial household bat roosts to collect and sell guano as fertilizer. We investigated farming practices and attendant spillover risks using (1) surveys on guano production; (2) an estimation of bat population size and species present using carcasses, visual identification, and audio recordings; (3) surveys of guano-producing and neighboring households on water, sanitation, and hygiene practices; and (4) the testing of guano and household food, water, and surfaces for coronaviruses using RT-qPCR. Bat roosts are constructed using dried palm leaves with coconut tree and/or steel/concrete supports. Roosting areas ranged from 42 to 327 m^2^, bat abundance varied from 0 to 11,187, guano production was between 5 and 120 kg/week, guano yields were from 0.15 to 0.4 kg/m^2^/week, and farmers earned USD ~100–200/household/month. Higher guano production in the peak (normally wet) season was associated with greater bat abundance (*p* = 0.016). The lesser Asiatic yellow house bat (*Scotophilus kuhlii*) was the only bat species identified. Roosts were <20 m from guano-producing households. Neighbors and households’ hygiene risks included not having handwashing stations and not covering food in storage/while drying. Coronaviruses (Alphacoronaviruses or Infectious Bronchitis Virus) were detected in 14.6%, 17.3%, 2.9%, 1.4%, and 0.0% of guano, urine, household surface, food, and water samples, respectively. While guano farming offers economic benefits, spillover risks exist. Safe guano collection and storage, handwashing, and food covering in guano-producing communities are necessary to mitigate spillover risks.

## 1. Introduction

Bat guano is commonly used as an organic fertilizer and is rich in carbon, nitrogen, minerals, and microbes [1,2]. Valued at anywhere between USD 1.25 and USD 12 per pound [2,3], bat guano is found in and harvested from caves [1,4,5]. This practice is common in Cambodia, where 37 of the 74 known bat species are found in caves [4,6], and bat guano harvesting has been documented in at least 38 caves [4]. Beyond caves, in the Mekong Delta region of Cambodia and Vietnam, farmers construct artificial roosts to attract free-ranging bats and collect guano [7]. These artificial roosts, often made from locally sourced materials such as palm fronds and supported by coconut trees or poles, are a common source of income for farming households. However, detailed studies on the specific methods, bat species involved, and associated zoonotic risks in these systems remain limited.

Zoonotic disease risks are inherent in human–bat interactions during guano harvesting [7]. In commercial harvesting from caves, guano harvesters may be exposed to certain hazards, including animal bites, dust inhalation, and infectious diseases [8]. This has been documented in Uganda, where bat exposure among those living near bat roosts was positively associated with the male sex, living in urban settings, hunting, and the perception of guano as a safe fertilizer [9]. These risks are exacerbated by poor knowledge among guano farmers. In Uganda, about 43% of participants thought guano was a safe fertilizer option [9]. Among guano miners in Thailand, 36% reported doing nothing or not knowing what to do in the event that a bat bit them [10]. In formative research for this study, bat guano farmers in Cambodia were found to have limited awareness of zoonotic risks and employed suboptimal biosafety measures, such as handling guano without any personal protective equipment (PPE) and having a lack of adequate water treatment and hygiene practices (unpublished data). These findings are consistent with past research that documents close interactions between communities and cave-roosting bats in Northwest Cambodia [11]. Thus, there is a need to understand the spillover risks of having bats close to households and communities.

Cambodia is home to at least 80 bat species [12], including *Mops plicatus*, *Taphozous* spp., *Hipposideros larvatus*, *Rhinolophus microglobosus*, *Hipposideros armiger*, and *Rhinolophus shameli* [4]. Bats are reservoirs for viruses such as coronaviruses, Nipah virus, and astrovirus, which have the potential to spill over into human populations [13]. Nipah virus infection has been detected in Cambodian bat populations, though no evidence of human cases has been recorded [14]. Coronaviruses are also common findings in Cambodian bats, with positive polymerase chain reaction (PCR) detection in 4.2% of cave-dwelling bats in Kampot Province and 4.75% of flying foxes in Kandal Province in Cambodia [15]. Coronavirus shedding was most frequent among juvenile and immature Cambodian bats [15]. The transmission of RNA viruses, particularly coronaviruses, can occur between bat species, which creates further opportunities for viral evolution and spillover [16]. There is a deep reservoir of coronavirus diversity in Southeast Asian bats, much of it undescribed, and one of the closest known relatives of SARS-CoV-2 was detected in *R. shameli*, known as the horseshoe bat, from northern Cambodia [17].

Proximity between artificial bat roosts and households represents a risk factor for zoonotic spillover. Zoonotic pathogens could spread through direct contact with guano during harvesting, airborne exposure to particulates, or contamination of household food and water supplies [13]. Epidemics of coronaviruses such as SARS-CoV and Swine Acute Diarrheal Syndrome–Coronavirus have been attributed to bats in Southeast Asia [18]. Sánchez et al. [18] estimate that as many as 66,000 people are infected with SARS-related coronaviruses annually in South Asia. Living close to bat roosts puts farmers and their families, communities, and livestock at risk of such exposure. Beyond the impact on human health and spillover risk, there are also implications for economic development and health system capacity.

Despite advances in understanding zoonotic disease risks, significant gaps remain. While previous studies have identified a variety of viruses in free-ranging bat populations, the mechanisms of spillover and human infection pathways are not fully understood [19]. Additionally, sociocultural factors influencing guano-harvesting practices require further exploration to develop culturally appropriate interventions [11]. To fill these research gaps, we conducted four related activities to understand guano farming practices and spillover risks: (1) surveys with guano farmers focused on production and farming; (2) estimates of bat population size and the determination of species using biometrics and visual and audio recordings; (3) surveys of guano-producing and neighboring households on water, sanitation, and hygiene (WASH) practices; and (4) the testing of guano, urine, and household food, water, and surfaces for coronavirus RNA. The goal of our research was to describe farming practices and identify spillover risks to enable the development of interventions to reduce spillover risks as part of the larger STOP Spillover project conducted by Tufts University and partners.

## 2. Materials and Methods

This study was conducted in three communities located in the central lowlands of Cambodia in Kampong Cham Province. Kampong Cham has a population of 895,763 people [20], where most (51–53%) of the income is derived from agriculture [21]. This area was selected for study inclusion because it had been identified in previous STOP Spillover work as having households engaged in guano farming. Working with community leaders and local authorities, we identified 17 guano farms in these three communities for study inclusion. Data were collected in the form of farming practices surveys, direct observations of farmers, overnight observations of bats, sample collection on guano farms, household WASH surveys, and sample collection at guano-producing and neighboring households.

The farming practices survey questionnaire was designed to collect data on guano farming practices, including demographics; farm size; roost construction materials and style; roost management practices; bat species occurrence, abundance, and seasonality; historical and current guano production and sales; and beliefs about factors affecting bat populations. Bat roost structures were also photographed, sketched, and measured (height to lowest leaves, length, width) using a laser rangefinder (Leica, Wetzlar, Hesse, Germany). Trained and supervised enumerators conducted these approximately 30-min surveys with all 17 identified households engaged in guano farming. Survey data were collected using KoboToolbox (Cambridge, MA, USA) on a Samsung (Suwon, South Korea) tablet, with paper forms available for backup. Data were exported into Microsoft Excel (Redmond, WA, USA) for cleaning and descriptive analyses.

To estimate bat abundance, a wired digital video recorder (DVR) camera system (Zosi Technology, Hong Kong) was installed for one overnight observation per farm. This system included four infrared-enhanced video cameras, a DVR recorder, a 12V lead-acid battery, and a voltage regulator. System set-up was completed before the evening emergence period, when bats exit the roosts in search of food [22]. To avoid double-counting and investigate the peak emergence period, counts were made for one hour from the first observed bat emergence. The first and last bat departure times (and any returns) for each camera within the first hour were also noted. Lastly, images and video of the entire guano farm were used to estimate the proportion of the perimeter covered by the camera system. In the data analysis, this proportion was used to correct the observed bat numbers by dividing the count by the estimated proportion under observation. To estimate bat species, data were collected concurrently with survey visits and with bat abundance estimation. Data from these investigations were triangulated to compare the results and identify bat species present.

**Figure A1:**
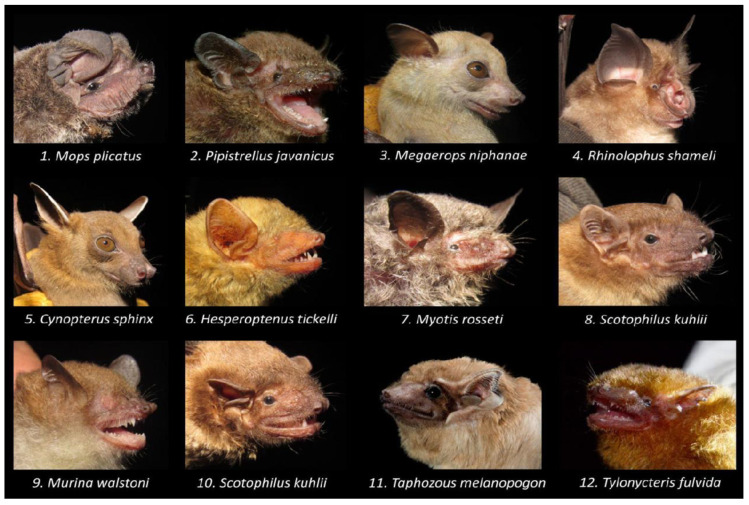
Portraits of Cambodian bat species employed in interviews. Image credits: Neil Furey (images 1–10, 12), Merlin Tuttle (image 11).

During farmer survey visits, opportunistic searches were undertaken for bat carcasses underneath roost structures. If found, these were photographed, and their forearm lengths were measured with a digital vernier caliper from the extremity of the elbow to the extremity of the carpus with the wings folded. Species were identified in accordance with standard morphological criteria [23,24]. To obtain an additional data point and better understand farmers’ knowledge, a placard with 12 unlabeled images of 11 bat species was presented (Figure A1). Each respondent was asked to select species that they believed were present on their farms.

During overnight observation, acoustic data were collected at each farm using Song Meter 4 full-spectrum bat detectors fitted with calibrated U2 ultrasonic microphones (Wildlife Acoustics, Maynard, MA, USA). A single device was deployed for one night at each farm and programmed to record from 30 min before sunset until sunrise. Acoustic data were analyzed through the visual inspection of recordings (via call frequencies, structure, and duration) in Adobe Audition (Adobe Systems, San Jose, CA, USA) and Batsound (Pettersson Elecktronic, Uppsala, Sweden). Identifications were made to the lowest taxonomic level possible based on datasets of verified recordings for known bat species from Cambodia (Neil Furey, personal correspondence).

Deviations from normal distribution were tested using the Shapiro–Wilk Test. Estimated bat abundance was log-transformed when the assumption of normality was violated. The correlation of pairs of normally distributed variables were assessed using linear regression. Differences in proportions between groups were assessed using Fisher’s Exact Test. Statistical analyses were performed using R (R Foundation for Statistical Computing, Vienna, Austria).

For bat guano collection, before dawn in the overnight observations, a 1 m^2^ plastic sheet for every 10 m^2^ of occupied roosting area was placed under roosts to non-invasively collect guano and urine samples. Two guano samples and one urine sample per sheet were collected. After 15 min, fecal samples were scooped up using plastic spoon-tip straws, and urine was collected by absorption onto sterile polyester swabs (Copan Diagnostics, Murrieta, CA, USA). Both sample types were then placed into a 2 mL cryovial (CryoKING, Biologix Group LTD, Changzhou, China) containing 0.9 mL of DNA/RNAShield (Zymo Research, Irvine, CA, USA) and kept on ice until reaching the laboratory, where they were kept at −20 °C until testing.

Regarding food, water, and surface survey and sample collection, data were collected from 10 randomly selected guano farms and the 10 nearest adjacent neighbor households. The number of households sampled was determined based on laboratory capacity, funding, and time availability, as well as the expected number of households required to document variation in household practices. Enumerators received training on informed consent, personal protection, survey implementation, and standard operating procedures for surface, food, and water sampling. Concurrently, dedicated enumerators conducted approximately 60-min interviews with households related to food, water, and surface practices while other dedicated enumerators wrote down a map of the households and selected approximately 10 samples from surfaces and food, as well as water samples of drinking and irrigation water. After surfaces were selected, an alcohol- and bleach-disinfected 10 × 10 cm stencil was placed on the surface, and a sterile polyester swab was swiped across the surface in horizontal, vertical, and diagonal directions. The swab was then placed in a tube prefilled with 0.9 mL of DNA/RNAShield. The tube was sealed and transported to the laboratory in a cooler with ice. Samples were stored at the laboratory at −20 °C until analysis. Duplicate water samples of 500 mL were collected from drinking water and irrigation water storage containers into Whirl-Pak^®^ bags (Pleasant Prairie, WI, USA) and stored on ice until reaching the laboratory.

All guano, urine, surface, food, and water samples were tested for the presence of CoV RNA at the Virology Unit, Institut Pasteur du Cambodge. Water samples were pre-processed by transferring 100 mL into a vacuum-driven filtration system using a Stericup^®^ (Millipore Sigma, St. Louis, MO, USA). Water was flowed through a 0.45 μm PVDF membrane filter supported by a filter support. The guano samples were first centrifuged (GT 422 centrifuge, 3000 rpm, 2 min, 4 °C) to remove any sample material from caps before being homogenized using a pellet pestle. Subsequently, samples were vortexed ~20 s and centrifuged. Food samples were homogenized in lysis buffer and centrifuged. All samples were then pooled, with five samples/pool (100 μL per individual sample). Pooled samples were then vortexed and centrifuged (3000 rpm, 15 min). The supernatant was recovered and filtered using a syringe filter with 0.45 μL pore size Nalgene Sterile Syringe Filters (Thermo Scientific, Waltham, MA, USA). RNA was extracted from each pool using the Zymo Research Direct-zol RNA MiniPrep kit (Zymo Research), and cDNA was transcribed using SuperScript III First-Strand Synthesis Super-Mix (Invitrogen, San Diego, CA, USA). Pan-CoV conventional hemi-nested RT-PCR targeting the RdRp gene was performed as previously described [25] in a 50 μL reaction volume.

All positive samples identified using pan-CoV conventional RT-PCR were subsequently sent for Sanger sequencing at Macrogen, Inc. (Seoul, Republic of Korea). Sequencing was performed in forward and reverse directions using primers from the second round of the hemi-nested PCR. The sequences obtained were confirmed for similarity using the National Center for Biotechnology Information (NCBI) nucleotide BLAST search (https://blast.ncbi.nlm.nih.gov/Blast.cgi, accessed on 8 March 2026). Positive pools were disaggregated and samples were run via the same protocol to yield individual results. The results of coronavirus testing were entered into Excel. Initial sequence analysis was performed using Geneious Prime 2022.1.1 (Biomatters Ltd., Auckland, New Zealand) and Blast Search (NCBI). The nearest 100 matches were recovered from Genbank (NCBI), and pairwise identity was used for comparison. A phylogenetic tree was built with Muscle 2.1 in Geneious Prime (Biomatters Ltd.) and generated via Neighbor-joining and the Tamura–Nei genetic distance model. The outgroup was set as SARSCoV-2 human USA.

## 3. Results

**Figure A2:**
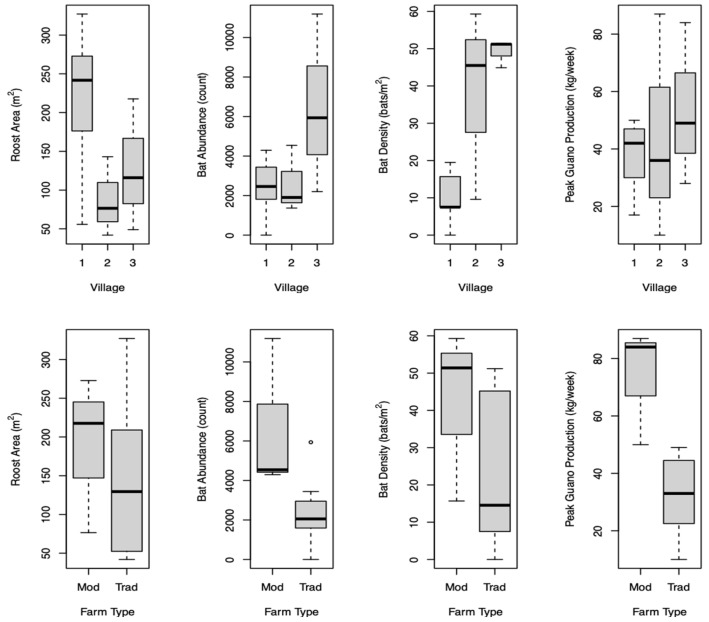
Box and whisker plots showing (from left to right) roost area, bat abundance, bat density, and peak weekly guano harvest from guano farms in Kampong Cham, Cambodia in 2023. Top row is separated by the three villages in the study area, and the bottom row by traditional (Trad) vs. modern (Mod) material roost types.

Guano farming surveys and roost measurements were completed at 17 farms, with bat abundance estimates and bat identification completed at 11 of these due to time constraints, equipment malfunctions, and limited funding (Table 1, Figure A2). A total of 480 guano samples (261 feces and 219 urine samples) from 17 guano farms were collected over four seasons, accounting for seasonal variations and the bat reproductive phase in pathogen detection. Food, water, and surface samples were collected from 10 guano farm households and 10 neighboring households and included 5211 samples (376 surfaces, 70 food samples, 75 water samples).

### 3.1. Farmer Surveys

Among 17 surveyed farms, most respondents (*n* = 16, 94%) had completed primary school, and just over half (*n* = 9, 53%) were women. The average age of the respondents was 59.8 years old, and the mean household size was 4.7. Most farms (*n* = 16, 94%) were located <20 m from the households (range 1-80m), reportedly to discourage theft and predation by snakes or owls. Men (*n* = 29, 49%), women (*n* = 22, 37%), boys (*n* = 5, 8%), and girls (*n* = 3, 5%) reported working in farming bat guano, with men and women spending <1 h/day working. Women (*n* = 16, 94%) were more frequently involved in packaging and drying guano, cleaning the farm, clearing away dead bats, and changing the nets to filter guano than men (*n* = 12, 71%). Men (*n* = 17, 100%) and women (*n* = 15, 88%) both participated in guano collection from under the roosts. The only task almost always performed by men (*n* = 16, 94%) was changing bat roost leaf bunches. Seventy-six percent (*n* = 13) of surveys reported that women were responsible for decision-making on roles and responsibilities at the farm, 41% (*n* = 7) for guano price, and 53% (*n* = 9) for interacting with buyers. Nearly all farmers (*n* = 16, 94%) reported their primary income was guano farming, and almost all (*n* = 16, 94%) reported earning between USD 101 and 200/month from farming.

**Figure 1:**
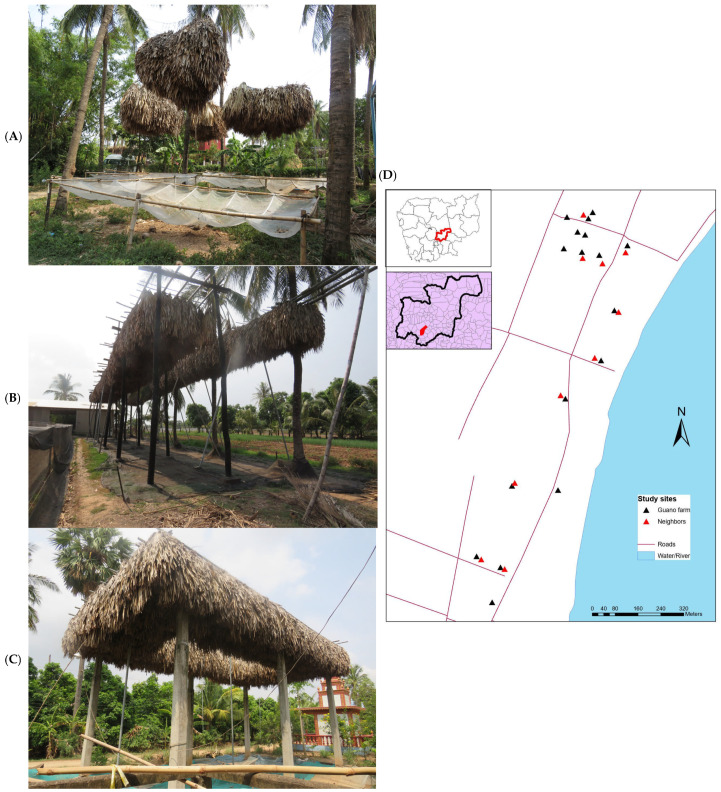
(**A**):Traditional dome roost in Kampong Cham Province, Cambodia, (**B**): traditional linear roost in Kampong Cham Province, Cambodia, (**C**): modern roost on artificial supports in Kampong Cham Province, Cambodia. (**D**): Locator map showing study sites in Kampong Cham Province, Cambodia.

In all farms, the roosts were composed of dried sugar palm (*Borassus flabellifer*) leaf bunches suspended from supports (Figure 1). Six farms had traditional coconut tree roosts (three dome-like and three linear). Eleven farms had modern (steel/concrete support) roosts. The respondents reported that guano farms are only successful in areas with naturally occurring bat populations. Farmers reported originally building roosts to collect bat guano for use as fertilizer in family vegetable gardens. Later, to respond to the demand for natural fertilizer, the bat guano businesses were expanded, commercialized, and passed from one generation to the next. All seventeen (100%) farms surveyed used plastic netting to collect guano, and eleven farms (65%) used raised nets above ground level and six (35%) kept nets at ground level. The respondents reported changing leaf bunches every other year to reduce parasite loads and avoid bats abandoning the roosts.

Farm roosting areas ranged from 42 to 327 m^2^ and production ranged from 5 to 49 kg/week during the dry season, with a peak output of 10–120 kg/week during the wet season. The mean yield of guano varied seasonally between 0.15 and 0.4 kg/week/m^2^ of roost area. According to the participants, guano typically sells for USD 1.25/kg. Most producers collect between two and three bags/week, weighing 20–22 kg/bag, with a total value between USD 50 and 75/week.

### 3.2. Bat Species and Behavior

**Figure A3:**
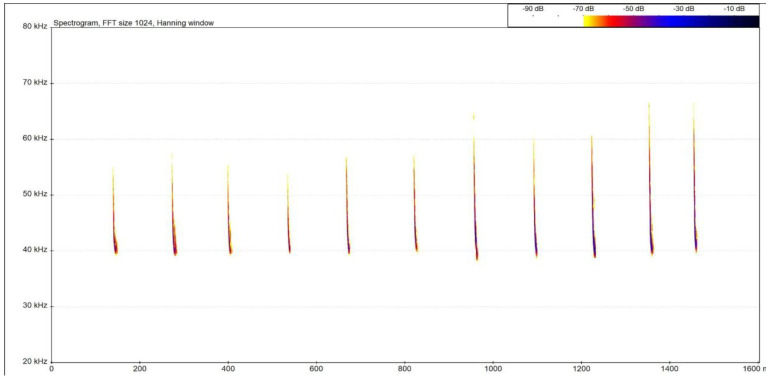
Example spectrogram of search phase calls emitted by *S. kuhlii* collected at a bat guano farm in Kampong Cham, April 2023.

A total of seven carcasses were found on six of the seventeen farms (35%). All carcasses were identified as *Scotophilus kuhlii* based on morphology. Acoustic recordings were obtained at 14 farms, and analysis confirmed all but a small minority of signals closely matching those emitted by *S. kuhlii* (Figure A3). Based on the image lineup of bat species (Figure A1) shown to respondents, twelve (80%) identified an image of *S. kuhlii*, while three (20%) selected an image of *Murina walstoni*, a forest-dwelling species not known from this region of Cambodia.

In general, bats began foraging flights around 18:00 (10–20 min after sunset) and began returning at 21:00–22:00. There was continuous traffic in and out of roosts during the night. All bats were observed returning by 04:00–05:00. The timing and direction of the emergence flights was accurately predicted by farmers. Farmers reported female bats give birth to up to two pups per year in April/May and believe deaths are caused during pregnancy, when young bats learn to fly, during hot weather, and in heavy rains.

Estimated bat abundance varied between zero (unoccupied sites) and 11,187 individuals, with an average of 3558. Roost area and estimated bat abundance were not significantly associated (*p* = 0.449). Peak bat guano production was strongly associated (*p* = 0.016) with estimated bat abundance.

### 3.3. Household Survey

The survey was conducted with 10 guano-farming and 10 neighboring households. Most respondents were female (*n* = 19, 74%), with an average age of 56.7 years. Literacy rates were high, with 80% (*n* = 20) of female and 89% (*n* = 19) of male heads of households being able to read, and 80% of all respondents having attended school to at least primary school level (*n* = 20).

In the following results presentation, *n* = 10 for guano-farming and non-guano-farming households, unless otherwise mentioned. All households had access to wired electricity. House construction varied, with all guano-farming and 50% of neighboring households having wooden walls, and 80% of guano-farming and 60% of neighboring households having dirt floors. Additionally, 80% of guano-farming and 70% of neighboring households had tile roofs.

Household taps were the primary source of drinking water for 80% of guano-farming and 50% of neighboring households. Protected wells were used for non-drinking water by 40% of all households. Most respondents (100% of guano-farming and 80% of neighboring) reported covering their water storage containers. Water treatment practices varied, with 70% of guano-farming households reporting treating their water compared to 50% of neighboring households, primarily through boiling. All households used water for vegetable cultivation, with 70% of guano-farming and 40% of neighboring households covering their water storage containers used for irrigation. Overall, 30% of guano-farming and 40% of neighboring households used refrigerators for food storage, with additional storage options including bags and cool boxes. Notably, 80% of both guano-farming and neighboring households dried food or meat outside without covering it.

Overall, 90% of households had dedicated cooking spaces, and most respondents (90% farming, 70% non-farming) also had designated dishwashing areas. Soap and sponges were commonly used for cleaning dishes, and 70% of all households used wet mops with detergent to clean their floors. Individual household latrines were used by 80% of guano-farming and 100% of neighboring households, and all respondents reported using soap to clean latrines and wash their hands. However, only 20% of guano-farming households had dedicated handwashing stations (with 0% of neighboring households having this), and only 20% of respondents used running water for handwashing. Trash was stored in bags and burned by all households.

All guano-farming and 60% of neighboring households were located within 20 m of bat roosts. Disturbances from bats, such as entering the house, water contamination, bat excreta, and bat noise, were reported by 10% of neighboring households. In total, 346 surfaces, 70 food samples, and 75 water samples were collected from the 20 households. The substrates sampled included bat roost structures, baskets over food, ceramic containers for bat guano storage, clothes (near bat roosts and in and outside of houses), cooking tables, covers on rice, hats for collecting bat guano, outside tables, plates (inside in kitchen uncovered and outside kitchen), railings, tables, fridges, kitchen tables, toilet doors, upstairs floor, upstairs table, and water containers (outside and in the kitchen). Surface samples from food included dried bananas, coconut waste, dried fish, dried pork, green vegetables (leftovers), jackfruits, leftover fish, leftover pork, mangos, oranges, potatoes, raw meat, rice, sugar cane, tomatoes, and vegetable waste or garbage. Water samples were collected from drinking water and irrigation water storage containers.

### 3.4. RT-qPCR Testing Results

A total of 38 fecal samples (14.6%) and 19 urine samples (17.3%) tested positive for coronaviruses (Table A1 and Table A2). The overall positivity rate of both fecal and urine samples was 15%, but the average positivity percentage by farm ranged from 0 to 50% (with higher percentages in small farms). Sample-level positivity was higher during the rainy season (21.7%) compared to the dry season (12.4%), and this difference was statistically significant (*p =* 0.030). Significant differences (*p =* 0.01247) in sample-level positivity were also observed based on bat reproductive periods. Samples collected in March at the bat prenatal phase had a sample-level positivity of 24.3%. This declined to 17.2% in the bat pupping phase (April–May), 5.7% in the juvenile phase (August), and 2.7% in the adult phase (December).

**Figure 2:**
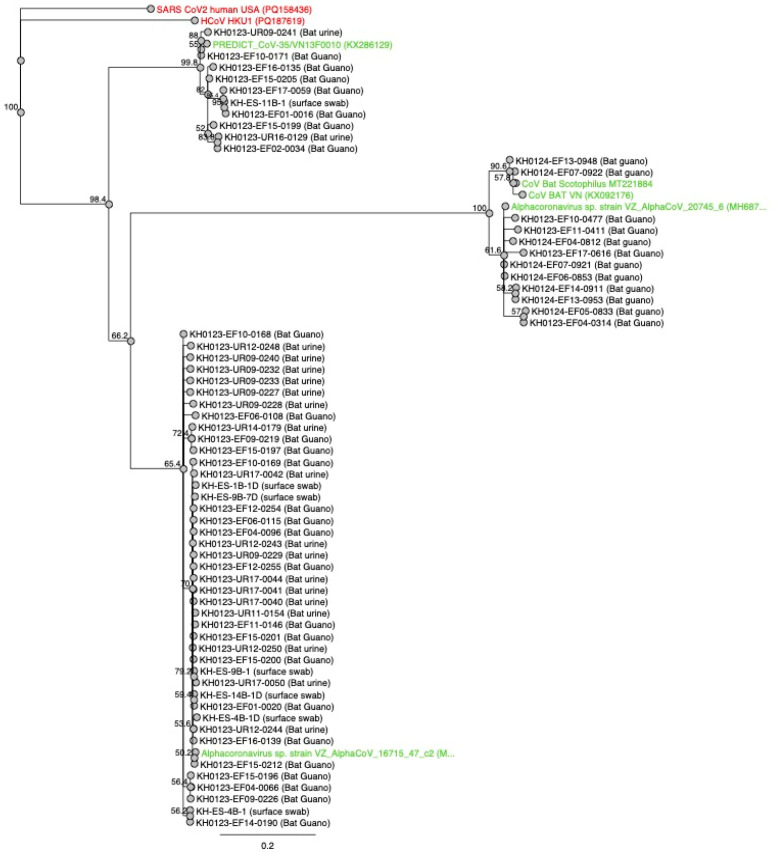
Maximum likelihood phylogenetic tree of coronavirus samples obtained from bat guano, bat urine, and surface swab samples (black) collected from guano farms in Kampong Cham Province, Cambodia, in 2024 based on a ~294bp region of the RdRp gene. Reference sequences are shown in green (bat-origin alphacoronaviruses) and red (human betacoronaviruses). Tree was fitted with Neighbor-joining and the Tamura–Nei genetic distance model. The outgroup was set as SARS-CoV-2 human USA. The nearest 100 matches were recovered from Genbank (NCBI) and pairwise identity was used for comparison.

Positive results of either alphacoronaviruses or Infectious Bronchitis Virus (IBV) were detected in 11 (2.9%) of the 376 surface samples. Alphacoronaviruses were detected in seven samples from guano-farming households. Six positive samples were from bat roost surfaces collected from five of the ten (50%) guano-farming households, and one sample was an outside table surface collected from one guano-farming household (10%). IBV was detected on four household surfaces, including food covers at one guano-farming household and a kitchen table, outside table, and upstairs table at neighboring households. IBV was detected in one (1.4%) of seventy food samples, specifically from a coconut surface collected at a guano-farming household. No viruses were detected in the 75 water samples. Phylogenetic analysis of these samples showed that all positive samples from bat guano were closely related to uncharacterized alphacoronaviruses previously detected in bat guano and/urine samples from Southeast Asia (Figure 2). Positive surface samples contained either similar alphacoronaviruses or IBV.

## 4. Discussion

To our knowledge, our research is unique in examining the spillover risks of artificial bat roosts to produce bat guano as fertilizer. We employed a range of methods to understand the methods of guano production and exposure risks to potential zoonotic viruses associated with guano farming. The findings revealed that bat guano farming is a lucrative enterprise, and farmers are highly knowledgeable regarding the bats they work alongside. Additionally, while many positive WASH practices were common in households, areas for improvement exist around water treatment, food storage, and handwashing. Lastly, risks of spillover exist as evidenced by the detection of bat-associated coronaviruses not only in guano and urine (15%), but also, though less commonly, on household surfaces (2.9%) and food (1.4%), though not from water samples (0%). We also discuss recommendations and conclusions from this research.

Bat guano farming is a lucrative enterprise and serves as the primary source of income for many producers. Guano farmers kept detailed records of production and reported earning about USD 1.25/kg of fertilizer, resulting in a monthly income between USD 101 and 200. These economic indicators are consistent with previous findings [2,3]. Farmers had a high degree of knowledge of their farms, including accurate predictions of bat species and the timing and direction of emergence flights. Previous researchers have demonstrated variable levels of understanding and knowledge of bats among people with different relationships to bats. A high degree of knowledge of bat behavior and ecology was evident in an ethnobiological study in Madagascar, which documented extensive knowledge of bat species among people who interacted with and collected guano from bats [26]. By contrast, farmers in Belize, whose principal interaction with bats was through conflict, exhibited minimal knowledge of ecosystem services provided by bats [27].

Bat guano farms are often passed down within families, indicating that they are a valued cultural and/or economic asset. This aligns with research from other parts of the world, where participants reported positive perceptions of bats due to their provision of guano, the ability to enhance tourism and control pests, and their potential use as a food source [27]. Despite these positive associations, there were some areas of concern identified in this study, including the following: (1) bat roosts are located in close proximity to households and neighbors, exacerbating any potential health risks; (2) there was low risk perception and low use of PPE among farmers, which aligns with previous research in Uganda and Vietnam [9]; and (3) gender disparities were reported in guano preparation tasks, which may result in concentrated exposure within specific demographics, particularly older women.

WASH practices within farming households were generally positive, with most households covering water storage and using either treatment or other safe water sources for drinking. Most households had designated cooking and dishwashing areas, used soap and detergent, and had access to latrines. However, most households lacked dedicated handwashing stations and had poor food storage practices, including not covering food indoors or while drying. This aligns with another study in Cambodia, where 95% of households did not have handwashing stations and minimal handwashing occurred at key times [28]. Handwashing before and after cooking has also been found to be positively associated with rural farming households’ knowledge of zoonoses in Cambodia [29].

Bats are well known as natural reservoirs of many zoonotic viruses, including SARS-CoV, indicating the potential for spillover risk in bat–human interfaces [15,30,31,32,33]. Our study found the highest frequency of coronavirus positive samples in bat guano and urine, followed by surfaces and food. Previous research in Vietnam showed that as many as 76.5% of bat guano samples were positive but detected no positives in urine samples [7]. In Sarawak, East Malaysia, 40% of bat guano samples tested positive for coronaviruses, including both alpha and betacoronaviruses [19]. Of guano samples collected in a recent study in Myanmar, 25.8% were positive for a coronavirus [34]. This variability illustrates the dynamic nature of disease transmission in bats and is also likely affected by non-standardized methods for sampling and processing guano for coronavirus testing. The potential for cross-contamination between guano and urine is also generally unquantified, and this may influence these results in either direction. Sample positivity rates can also be heavily impacted by UV degradation depending on environmental conditions. Though there is much variability among viruses and bat species, guano has been established as a valuable non-invasive tool for detecting bat-borne viruses.

The viruses detected in this study do not differ markedly from those found in previous work. Alphacoronaviruses have been detected in *S. kuhlii* in Cambodia and Vietnam [35]. As with this previous work, no betacoronaviruses and no viruses with known public health significance were detected. The finding of IBV was likely attributable to the frequent presence of chickens on farms. Although likely unrelated to the presence of bats, IBV is also a coronavirus, and the detection of IBV RNA on household surfaces demonstrates the potential for the transfer of coronaviruses around farms and into households by farmers and their families.

Our results showed a distinct seasonality of virus shedding in bats, with the greatest sample positivity occurring across the perinatal period. A wide range of seasonal patterns of viral shedding has been recorded in bats, but some, including for some coronaviruses, have shown a similar pattern with greatest shedding occurring around pupping [36]. The shedding of coronaviruses by bats in Cambodia and Thailand showed a pattern of maternal–juvenile transmission during the perinatal period followed by a pulse of increased shedding prevalence among juveniles [15,37]. The coronavirus shedding patterns of *Mormopterus francoismoutoui* in Réunion Island were very similar to those seen in our study, with regular seasonal peaks in coronavirus shedding from just before to just after pupping [38]. While the exact patterns of the shedding of specific viruses by host species vary, it is common to find seasonal patterns that align with specific life stages of the host.

The spillover risks identified in our study underscore the need for risk reduction programs. Local spillover risk is driven by the frequency, duration, and intensity of contact between human hosts and wildlife reservoirs [39]. This study indicates clear evidence of close, frequent contact with bats on and surrounding bat guano farms. Specifically, our findings support the importance of interventions targeting hygiene (e.g., food storage, handwashing) and PPE for farmers to reduce spillover risks for guano farmers, their neighbors, and local communities.

This study had several limitations, namely a small sample area, cross-sectional design, and challenges analyzing samples. All samples came from one region in Cambodia, which limits our understanding of zoonotic disease transmission and spillover risks in other areas of Cambodia and in surrounding countries, such as Thailand and Vietnam. The cross-sectional design does not allow for the investigation of longitudinal patterns in disease transmission, particularly as it relates to seasonality. Lastly, there were challenges in analyzing samples, especially in local laboratories that found sourcing materials for testing difficult. We recommend future research on the role of seasonality in spillover risk, actual viral risk, and evaluations of the effectiveness of culturally appropriate interventions (especially PPE, handwashing, and food storage) to reduce the risk of spillover in farmers and their communities.

Overall, while guano farming offers economic benefits, spillover risks exist. Safely collecting and storing guano, handwashing, and covering foods in guano-producing communities are necessary to mitigate spillover risks. Further research should examine seasonality, attempt to better characterize zoonotic viral risks, and assess intervention effectiveness to ameliorate our understanding and prevent spillover in Cambodia and beyond.

## Figures and Tables

**Table 1:** Sampling framework.

Sample Type	Quantity	Collection Date
Farmer survey	17 farms	April–May 2023
Household and neighbor WASH survey	20 farms	April 2023
Bat abundance	11 farms	April–May 2023 August 2023 December 2023 March 2024
Bat species identification	7 carcasses (6 farms)	April–May 2023
Bat feces samples	261 samples (17 farms)	April–May 2023 August 2023 December 2023 March 2024
Bat urine samples	219 samples (17 farms)	April–May 2023 August 2023 December 2023 March 2024
Household surface samples	376 samples (10 farms)	April 2023
Food samples	70 samples (10 farms)	April 2023
Water samples	75 samples (10 farms)	April 2023

## Data Availability

The original contributions presented in this study are included in the article. Further inquiries can be directed at the corresponding author.

## Abbreviations

The following abbreviations are used in this manuscript:

IBV	Infectious Bronchitis Virus
NCBI	National Center for Biotechnology Information
PPE	Personal protective equipment
PCR	Polymerase chain reaction
WASH	Water, sanitation, and hygiene

## Appendix A

**Table A1:** Positivity rate of individual farm by sample type.

ID Farm	Positive Samples of Feces and Urine N (%)		
	Feces	Urine	Total
1	2 (0.13)	0 (0.00)	2 (0.07)
2	1 (0.08)	0 (0.00)	1 (0.05)
3	0 (0.00)	-	0 (0.00)
4	5 (0.14)	0 (0.00)	5 (0.10)
5	1 (0.13)	-	1 (0.13)
6	3 (0.12)	0 (0.00)	3 (0.09)
7	2 (0.33)	-	2 (0.33)
8	0 (0.00)	-	0 (0.00)
9	2 (0.12)	7 (0.44)	9 (0.27)
10	3 (0.17)	0 (0.00)	3 (0.13)
11	2 (0.09)	1 (0.14)	3 (0.10)
12	2 (0.13)	4 (0.40)	6 (0.24)
13	2 (0.50)	-	2 (0.50)
14	2 (0.25)	1 (0.20)	3 (0.23)
15	7 (0.25)	0 (0.00)	7 (0.21)
16	2 (0.13)	1 (0.11)	3 (0.13)
17	2 (0.11)	5 (0.45)	7 (0.24)
Total	38 (0.146)	19 (0.173)	57 (0.154)

**Table A2:** Virus strains found in feces and urine in bat guano farm.

No.	Virus Strain	Frequency
1	Alphacoronavirus sp. strain VZ_AlphaCoV_16715_47_c2, complete genome	18
2	Alphacoronavirus sp. strain VZ_AlphaCoV_16715_56, complete genome	1
3	Alphacoronavirus sp. strain VZ_AlphaCoV_16715_61, complete genome	3
4	Alphacoronavirus sp. strain VZ_AlphaCoV_16715_63, complete genome	2
5	Alphacoronavirus sp. strain VZ_AlphaCoV_16715_7, complete genome	9
6	Alphacoronavirus sp. strain VZ_AlphaCoV_16715_77, complete genome	2
7	Alphacoronavirus sp. strain VZ_AlphaCoV_16715_86, complete genome	3
8	Alphacoronavirus sp. strain VZ_AlphaCoV_16845_47, complete genome	13
9	Alphacoronavirus sp. strain VZ_AlphaCoV_17819_22, complete genome	1
10	Coronaviridae sp. isolate 75-55-L06-R2-CoV-BAT-VN RdRp gene, partial cds	1
11	Coronavirus PREDICT CoV-35 PREDICT_CoV-35/VN13F0010 F3R RNA-dependent RNA polymerase mRNA, partial cds	2
12	Scotophilus bat coronavirus 512 2005/PREDICT_VN13F0043 RNA-dependent RNA polymerase mRNA, partial cds	1
13	Scotophilus bat coronavirus 512 2005/PREDICT_VN13F0204 F1R RNA-dependent RNA polymerase mRNA, partial cds	1
Total		57
